# Intrahepatic cholestasis of pregnancy and associated causes of death: a cohort study with follow-up of 27–46 years

**DOI:** 10.1186/s12905-018-0606-0

**Published:** 2018-06-19

**Authors:** Suvi-Tuulia Hämäläinen, Kaisa Turunen, Kari J. Mattila, Markku Sumanen

**Affiliations:** 1 Department of General Practice, Faculty of Medicine and Life Sciences, University of Tampere, 33100 Tampere, Finland; 2 Janakkala Health Centre, Tapailanpiha 13 B, 14200 Turenki, Finland; 3 Science Center, Tampere University Hospital, 33521 Tampere, Finland

**Keywords:** Intrahepatic cholestasis of pregnancy, Causes of death, Mortality

## Abstract

**Background:**

The aim of this study was to determine whether intrahepatic cholestasis of pregnancy (ICP) is associated with causes of death during on average 35 years follow-up after the delivery.

**Methods:**

The study population comprised 571 women with ICP in at least one pregnancy seen at Tampere University Hospital, Finland, between 1969 and 1988. ICP was verified from patient records. The previous and following subjects in the maternity ward diary were taken as controls for each ICP case. In total, there were 1333 controls. All underlying causes of death were obtained from Statistics Finland in March 2017. The deaths occurred during 1971–2015 and the causes of death were classified according to ICD-10.

**Results:**

Altogether, 39 of the mothers with ICP (6.8%) and 111 of the controls (8.3%) had died by the end of 2015 (*p* = 0.267). There were more underlying causes of death from gastrointestinal diseases (15%) in the ICP group than in the control group (4%) (*p* = 0.011). The number of underlying causes of death due to diseases of the circulatory system were lower in the ICP group (13%) than in the control group (26%), although the finding was not statistically significant (*p* = 0.088). Moreover, neoplasms were the underlying cause of death in 46% of cases among mothers with ICP and in 41% of cases among the controls (*p* = 0.609). Diseases of the other organ systems were rare in both groups.

**Conclusion:**

Women with a history of ICP do not have an increased overall mortality. However, deaths from gastrointestinal diseases are overrepresented among women with a history of ICP.

## Background

Intrahepatic cholestasis of pregnancy (ICP) is a reversible liver dysfunction during pregnancy. It is characterized by otherwise unexplained pruritus, especially on the palms of the hands, soles of the feet, and the abdomen. The diagnosis also requires a rise in serum bile acids and transaminases [1]. ICP is associated with perinatal complications, for example meconium staining of the amniotic fluid, stillbirth and preterm delivery [2, 3]. Ursodeoxycholic acid is the primary treatment used for ICP [4]. The mother’s ICP symptoms usually resolve within 48 h of delivery and biochemical abnormalities resolve within 2–8 weeks [5].

Mutations of several genes may influence the pathogenesis of ICP [6–13]. In addition, hormonal factors, especially estrogen and progesterone, may be involved in the pathogenesis of ICP [14]. Genetic predisposition to ICP is revealed by family clustering, the presence of ethnic and geographic variations, and mutations in gene coding for hepatobiliary transport proteins [1, 14]. Intrahepatic cholestasis has been associated with hepatobiliary cancers, some autoimmune diseases, and cardiovascular diseases [15]. Also increased risk for gestational diabetes and pre-eclampsia has been reported among women with a history of ICP [16, 17].

To examine factors influencing causes of death, a long follow-up is required. Due to the genetic background and higher occurrence of some diseases, the objective was to determine whether ICP is associated with causes of death during on average 35 years follow-up after the delivery. To the authors’ knowledge, this is the first study to investigate this association over such a long follow-up time.

## Methods

### Data source and study population

The study population comprised all ICP pregnancies at Tampere University Hospital (TUH) between 1969 and 1988. Patients with ICP were identified in the hospital discharge register using diagnosis codes. From 1969 to 1986, ICD-8 was used at TUH. Because ICD-8 did not include a precise code for ICP, we checked all the obstetric codes that might contain ICP: 637.9 Toxicosis NUD, 639.00 Pruritus, 639.01 Icterus gravis, 639.09 Necrosis acuta et subacuta hepatis, and 639.98 Aliae definitae. Thereafter, we checked the written diagnosis behind the code, and if it referred to ICP, we included the case for further selection. ICD-9 was used between 1987 and 1988, and it contained the appropriate codes 6467A Hepatosis gravidarum and 6467X Hepatopathia alia. The diagnosis was verified from each patient record with the presence of the main symptom of itching and abnormal laboratory test results. At least one of the following was required at that time at Tampere University Hospital: ASAT > 35 U/l, ALAT > 40 U/l, or bile acids 6 μmol/l or more. The study population comprised 687 ICP deliveries.

The study population included some women with repeated ICP deliveries, and each of these women were studied as an individual case. The ICP group thus contained 575 women. Two controls for each ICP delivery were taken; these controls were the previous and the next subjects in the maternity ward diary. Altogether, there were 1374 controls. The groups were comparable regarding age, educational level, and body mass index [18]. Four women were ruled out from the ICP cases because of a missing personal identity code, as were 41 women from the controls. The final cohort comprised 571 women with ICP and 1333 controls. The groups were comparable regarding the participants’ age at labour, age at death, and age of those living at the end of 2015 (Table 1).

**Table 1:** Median ages of patients at labour, at death, and those still living at the end of 2015

	Mothers with ICP			Controls		
	n	age	age range	n	age	age range
At labour	571	27.6	16.9–41.1	1333	27.1	15.0–46.4
At death	39	56.7	34.1–78.5	111	55.9	28.1–83.8
Living at the end of 2015	532	63.7	48.0–80.8	1222	63.3	46.3–85.0

For the deceased in the cohort, we obtained the underlying causes of their death from 1971 to 2015 from Statistics Finland. The coverage of the underlying cause of death statistics is practically 100% [19]. The follow-up time of the cohort’s mothers was 27–46 years. The causes of death were classified using ICD-10 [20], and the older ICD-8 and ICD-9 codes were changed to the corresponding ICD-10 codes.

### Statistical analysis

The data were analysed using SPSS for Windows, version 22.0. The results are presented as frequencies and percentages. From cross tabulation, *p*-values were calculated using the Chi-squared test, and values of 0.05 or lower were considered statistically significant. Binary logistic regression analysis was performed to obtain odds ratio (OR) and 95% confidence intervals (CI). The dependent variable was “ICP or not”.

## Results

Altogether, 39 of the mothers with ICP (6.8%) and 111 of the controls (8.3%) had died by the end of 2015 (*p* = 0.267). The underlying causes of death are presented in Table 2. Each deceased has one underlying cause of death.

**Table 2:** Underlying causes of death among women with and without a history of ICP

Underlying cause of death (ICD-10)	Mothers with ICP *n* = 39		Controls *n* = 111		Difference	
	n	%	n	%	% units	*p*-value
Neoplasms (C00–D48)	18	46	46	41	5	0.609
Diseases of the digestive system (K00–K93)	6	15	4	4	11	0.011
External causes of morbidity and mortality, injury, poisoning and certain consequences of external causes (V01–Y98, S00–T98)	5	13	17	15	-2	0.705
Diseases of the circulatory system (I00–I99)	5	13	29	26	−13	0.088
Endocrine, nutritional and metabolic diseases (E00–E90)	2	5	5	5	0	0.874
Diseases of the nervous system (G00–G99)	2	5	5	5	0	0.874
Diseases of the musculoskeletal system and connective tissue (M00–M99)	1	3	1	1	2	0.420
Diseases of the respiratory system (J00–J99)	0	0	4	4	−4	0.230

The most common underlying causes of death were neoplasms, 46% of cases among mothers with ICP and in 41% of cases among the controls (*p* = 0.609). There were no hepatobiliary neoplasms in the ICP group and only one in the control group. Malignant neoplasms of digestive system were the underlying causes of death in 28% (*n* = 5) of ICP women and in 26% (*n* = 12) of controls (*p* = 0.890). In the ICP group the malignant neoplasms were colon (*n* = 2), small intestine (n = 1) and stomach (n = 2) cancers. Respectively, malignant neoplasms in the control group were pancreas (*n* = 3), ampulla of Vater (n = 1), colon (*n* = 6) and stomach (n = 2) cancers.

Diseases of the circulatory system were the underlying cause of death in 13% of cases among mothers with ICP and in 26% of cases among the controls (*p* = 0.088). Diseases of the circulatory system were the second common underlying cause of death among the controls.

Diseases of the digestive system were more often the underlying cause of death in the ICP group than in the control group (15% vs. 4%, *p* = 0.011). The risk to have a gastrointestinal cause of death was nearly 5-fold in ICP group compared to controls (OR = 4.85, 95% CI 1.29–18.18). Diseases of the digestive system were the second common underlying cause of death among mothers with ICP. Within gastrointestinal diseases, hepatobiliary diseases were the underlying cause of death among 67% (*n* = 4) of ICP mothers and 75% (*n* = 3) of controls (*p* = 0.778). Alcoholic liver diseases were found in four ICP mothers and in two controls and cirrhosis of liver in one control. Diseases of pancreas and alcohol-induced chronic pancreatitis were the underlying causes of death among two women with ICP. Gastro-oesophageal laceration-haemorrhage syndrome was the underlying cause of death in one control. In total, diseases of the digestive system and malignant neoplasms of the digestive system were the underlying causes of death in 28% (*n* = 11) of ICP women and 14% (*n* = 16) of controls (*p* = 0.054).

Diseases of the other organ systems were rare in both groups.

## Discussion

The main finding was that women with a history of ICP do not have an increased overall mortality. There were more deaths from gastrointestinal diseases in the ICP group than in the control group. Diseases of the circulatory system were twice as frequently the underlying cause of death in the control group than in the ICP group.

### Strengths and limitations

The data in our study were relevant and the methods were valid and reliable. One limitation of the study was the small number of death cases. Nevertheless, the follow-up time of this study was long, 27–46 years, comprising all ICP cases at TUH during the 1969–1988 period. A long follow-up time is required when investigating the causes of death of mothers. Although the follow-up time was long, the deaths represent those who died relatively young. Life expectancy of Finnish women at the age of 65 years was 21 years in 2014 [21]. The life expectancy of ICP mothers (*n* = 532) living at the end of the follow-up in 2015 is over 20 years. An extended follow-up time would show whether causes of death are the same for the mothers who die older.

A weakness of the study is that the levels of bile acids and/or transaminases used for diagnosis of ICP were somewhat lower than more recent criteria. There is a possibility of existing liver disease at the time of ICP diagnosis. However, the diagnosis of ICP requires exclusion of other reasons and the diagnosis was made by an obstetrician.

### Comparison with existing literature

It seems that the mothers with ICP die of cardiovascular diseases less frequently than expected and the controls more frequently than expected. The number of deaths from circulatory diseases seems to be lower in the ICP group, even though ICP has been associated with circulatory diseases [15]. On the other hand, women with ICP have reported less cardiac arrhythmia, high cholesterol, and high blood pressure requiring medication [22]. In 2015, diseases of the circulatory system were the underlying cause of nearly 38% of deaths among Finnish women over 15 years old [23]. In our study, diseases of the circulatory system were the underlying cause of death among 26% of the controls. Our cohort represents those who died younger, and the proportions of the causes of death might change if the cohort would be followed even longer. Although there were fewer deaths due to diseases of the circulatory system in the ICP group, it cannot be concluded that ICP protects from diseases of the circulatory system. ICP has been associated with gestational diabetes and pre-eclampsia [16, 17]. Gestational diabetes increases risk of type 2 diabetes, hypertension and ischemic heart disease [24]. An increased risk in cardiovascular diseases have been associated with pre-eclampsia [25]. Nevertheless, the mortality to circulatory system diseases was not higher in our study.

Despite the rather small number of deaths, statistically significant differences were found. The findings may be considered clinically relevant. Increased risk of hepatobiliary diseases has been reported among women who experience ICP [15, 22, 26, 27]. The increased occurrence of hepatobiliary diseases may be reflected in the increase of deaths from gastrointestinal diseases among the ICP group.

Hepatobiliary neoplasms as underlying causes of death were not detected in this study among ICP mothers. Previously an association between ICP and liver cancer and biliary tree cancer has been reported [15]. Nevertheless, the incidence of these cancers has been found to be 0.1% so that the expected number of these rare cancers in our cohort is rather low. In this study, we did not investigate the incidence of different cancers but the underlying causes of death in the cohort.

Risk consumption of alcohol was not remarkably different among ICP mothers compared to controls in a questionnaire study of the same cohort [28]. According to a large Swedish study alcoholic cirrhosis was less likely to be diagnosed with women with ICP compared to controls [27]. Alcohol consumption might have an effect on the underlying causes of death, particularly on hepatobiliary diseases. Women with a history of ICP have an increased risk of liver fibrosis and cirrhosis which might be caused by hepatitis C infection [24]. This finding may reflect on the increased risk of deaths from gastrointestinal diseases in ICP women.

Many genetic mutations have been found to be associated with ICP [6–13]. Our hypothesis is that ICP is a manifestation of a genetic background that exposes the individual to certain diseases and causes of death.

## Conclusion

Women with a history of ICP do not have an increased overall mortality. However, gastrointestinal diseases are overrepresented in the underlying causes of death among women with a history of ICP. This is the first study to examine the association between ICP and underlying causes of death over such a long follow-up time. An even longer follow-up time is required to investigate further the association between ICP and gastrointestinal causes of death. This would confirm whether the phenomenon found in our study continues among those who die older.
